# Metformin Changes the Relationship between Blood Monocyte Toll-Like Receptor 4 Levels and Nonalcoholic Fatty Liver Disease—*Ex Vivo* Studies

**DOI:** 10.1371/journal.pone.0150233

**Published:** 2016-03-01

**Authors:** Agnieszka Zwolak, Olga Słabczyńska, Justyna Semeniuk, Jadwiga Daniluk, Agnieszka Szuster-Ciesielska

**Affiliations:** 1 Department of Internal Medicine and Internal Medicine in Nursing, Medical University, Lublin, Poland; 2 Department of Endocrinology, Medical University, Lublin, Poland; 3 Department of Virology and Immunology, Maria Curie-Skłodowska University, Lublin, Poland; 4 Department of Gastroenterology with the Endoscopic Laboratory, Medical University, Lublin, Poland; Institute of Medical Research A Lanari-IDIM, University of Buenos Aires-National Council of Scientific and Technological Research (CONICET), ARGENTINA

## Abstract

**Background:**

Toll-like receptor 4 (TLR4) contributes to the development of NAFLD (nonalcoholic fatty liver disease) and MetS (metabolic syndrome). It is unclear whether anti-diabetic metformin affects TLR4 expression on blood monocytes, thereby protecting or improving inflammatory parameters. Therefore, we investigated TLR4 in patients with NAFLD meeting different sets of MetS criteria and linked the results with the disease burden.

**Methods:**

70 subjects were characterized and divided into three groups: (I) healthy individuals, (II) nonobese with NAFLD and without MetS, and (III) prediabetic, obese with NAFLD and MetS. We determined the concentrations of IL-1β, IL-6, TNFα, and monocyte TLR4 levels in fresh blood as well as in blood cultures with or without metformin supplementation.

**Results:**

The characteristics of the study groups revealed a significant association between NAFLD and BMI, MetS and inflammatory parameters, and TLR4. In ex vivo studies, 100 μM of metformin decreased the TLR4 level by 19.9% (II group) or by 35% (III group) as well as IL-1β and TNFα production. A stepwise multiple regression analysis highlighted a strong effect of metformin on attenuation of the link between TLR4 and NAFLD, and TNFα.

**Conclusion:**

We concluded that, by attenuation of the blood monocyte TLR4 level, metformin reduced their inflammatory potential—critical after recruitment these cells into liver. However, this finding should be confirmed after in vivo metformin administration.

## Introduction

Westernized diet and lifestyle are responsible for globalization of obesity—the main risk factor of co-morbidities such as nonalcoholic fatty liver disease (NAFLD), metabolic syndrome (MetS), cardiovascular disease (CVD), and cancer. According to the newest hypothesis, rather than being a mere “manifestation of the metabolic syndrome”, NAFLD is indeed a necessary precursor of the future development of MetS in humans [1]. Although closely associated with obesity, NAFLD develops among nonobese subjects as well [2]. Therefore, the earliest intervention is of particular importance in the case of obesity as well as NAFLD and MetS diagnosis.

It is estimated that 80%-90% of patients with fatty liver remain free of inflammation; however, NAFLD with MetS components may result in a sub-clinical- progressing to clinical inflammatory process referred to as nonalcoholic steatohepatitis (NASH) [3, 4]. Many signaling pathways have been described as a link between inflammation and metabolism with a prominent role of Toll-like receptors (TLRs). Among them, TLR4 has received the greatest attention as it is ligated with pathogens of gut microbiota [5]. Nonbacterial substances may also function as TLR4 ligands i.e. free fatty acids (FFAs) [6, 7]; however, some reports have indicated that FFAs do not activate TLR4 directly [8]. Both NAFLD and obesity are characterized by increased circulating endotoxin and FFA levels [8] as well as enhanced TLR4 expression on liver cells (mainly Kupffer cells) [5, 9] and blood leukocytes (mainly monocytes) [10]. Additionally, recent studies have demonstrated that progression of NAFLD to NASH is accompanied by recruitment and accumulation of blood-derived inflammatory cells in both adipose tissue and the liver [11, 12].

With its anti-hyperglycemic and anti-hyperlipidemic effect, metformin is commonly prescribed for the treatment of not only type 2 diabetes mellitus (T2DM) [13], but also MetS [14]. Another benefit is the anti-inflammatory effect of metformin manifested by a decrease in the production of IL-1β, IL-6, and TNFα [15, 16]. A very limited number of studies concern its ability to influence TLRs expression. In fact, up to now only two papers link attenuated TLR2 and TLR4 activity with protection of the infarcted heart in rats treated with metformin [17, 18].

Therefore, we hypothesized that metformin decreases TLR4 expression on blood monocytes in NAFLD patients (ex vivo studies). Moreover, its efficacy could be associated with the stage of patients’ disease and their phenotype status. To address this, in our studies we included subjects diagnosed as prediabetic, obese with NAFLD and MetS, nonobese with NAFLD and without MetS, and healthy individuals.

## Materials and Methods

### Study population

The participants in this study were recruited among patients admitted to the Medical University Hospital in Lublin (Poland) between April 2012 and December 2013. Information on medical history and lifestyle characteristics was obtained from all subjects by a questionnaire (S1 Table). From the group of interest, we excluded those with the presence of different potential causes of liver disease: (a) seropositivity for HBsAg or anti-HCV antibody, (b) daily alcohol consumption over 20g, (c) treatment with hepatotoxic, steatosis-provoking, or immunosuppressive drugs during the previous 6 months, (d) Wilson’s disease or haemochromatosis. Other exclusion criteria included T2DM, malignancy, clinical atherosclerosis, hematological or chronic kidney diseases, active infection, metformin treatment, and smoking. Finally, we chose 48 individuals, all Caucasians of Polish descent, who had never been treated due to liver diseases before. The control group comprised 22 healthy individuals undergoing a routine health check-up program. Informed written consent was obtained from all participants in this study, which was conducted according to the ethical principles stated in the Declaration of Helsinki and approved by the institutional review board at the Medical University in Lublin, Poland (Permit Number: KE-0254/112/2011).

### Laboratory and abdominal ultrasonography (AU) assessment

Anthropometric data were recorded from each individual. Venous fasting blood samples were drawn from all subjects to perform biochemical analyses, determine cytokine levels, measure the monocyte TLR4 level, and develop ex vivo studies. The laboratory test included glucose (mg/dl), HbA1C (%), total cholesterol (mg/dl) and its fractions (HDL, LDL (mg/dl), triglyceride (TG, mg/dl), aspartate aminotransferase (AST, IU/L), alanine aminotransferase (ALT, IU/L), high-sensitivity C-reactive protein (hs-CRP, mg/dl), and insulin (μl/ml). The degree of insulin resistance (IR) was determined by the homeostatic model assessment (HOMA-IR) with the formula: (glucose*insulin):22.5. Fatty liver was defined according to the results of abdominal ultrasonography (AU): increased echogenicity of the hepatic parenchyma with attenuation of the portal vein echogenicity. In six patients, the results of AU indicated advanced disease; therefore, liver biopsy was performed and such subjects were excluded from our study. We also calculated both the hepatic steatosis index (HSI) and the fatty liver index (FLI) according to formulas: HSI = 8*ALAT/ASPAT ratio + BMI (+2 if female) [19], FLI = (e^0.953^*^loge (triglycerides) + 0.139^*^BMI + 0.718^*^loge (GGT) + 0.053^*^waist circumference— 15.745^)/(1 + e^0.953^*^loge (triglycerides) + 0.139^*^BMI + 0.718^*^loge (GGT) + 0.053^*^waist circumference— 15.745^) * 100 [20]. HSI and FLI<30.0 ruled out while HSI≥36.0 and FLI ≥ 60.0 ruled in hepatic steatosis detected by AU. A simple index FIB-4 (below) was used to predict liver fibrosis and a need for liver biopsy. In patients, FIB-4 values between 1.45 and 3.25 indicate a low risk of liver fibrosis [21].

$$FIB4=\frac{Age\;\left(years\right)\times AST\;(U/L)}{Platelet\;Count\;\left(10^{9}/L\right)\times \sqrt{ALT\;(U/L)}}$$

Although the patient diagnosis was supported by the above indexes, the main limitation was the lack of liver biopsy in all participants.

Serum cytokine levels, IL-1β, IL-6, and TNFα were determined with commercially available kits (BD OptEIA, San Jose, CA, USA) according to manufacturer’s instructions. The minimum detectable concentration of IL-1β was 0.8 pg/ml, IL-6—2.2 pg/ml, and TNFα—2 pg/ml.

### Definition of metabolic syndrome and obesity

MetS was defined using criteria of the International Diabetes Federation/National Heart, Lung and Blood Institute/American Heart Association (IDF/NHLBI/AHA-2009)—the presence of three or more of the following features: 1. waist circumference ≥94 cm in men or ≥80 cm in women; 2. triglyceride level ≥150 mg/dl; 3. HDL-cholesterol level <40 mg/dl in men and <50 mg/dl in women; 4. systolic blood pressure ≥130 mmHg or diastolic pressure ≥85 mmHg; 5. fasting plasma glucose level ≥100 mg/dl. Prediabetes was defined by a fasting glucose level of 100–125 mg/dl and/or HbA1C 5.7–6.4% (American Diabetes Association) [22]. The body mass index (BMI) (kg/m^2^) was calculated for each person. According to the BMI definition given by the World Health Organization, Caucasian patients with BMI 18.5–24.99 were included in the normal range, and those with BMI≥30.0 were classified as obese.

Based on the IDF/NHLBI/AHA-2009 criteria of metabolic syndrome and the results of AU supported by calculation of HSI and FLI indexes as well as BMI, we finally selected and divided 48 patients into 2 groups: group II—nonobese patients with NAFLD and without MetS (two of the five IDF/NHLBI/AHA-2009 criteria) (n = 20); group III—prediabetic, obese subjects with NAFLD and MetS (meeting all IDF/NHLBI/AHA-2009 criteria) (n = 28). Healthy individuals served as a control group (I) (n = 22).

### Ex vivo study design

We investigated the production of IL-1β, IL-6, TNFα, and TLR4 level on monocytes of patients’ peripheral blood from the patients examined. Briefly, blood samples were collected in sterile heparinized tubes, diluted 1:4 with RPMI 1640, and cultured for 24 h with or without metformin (20 or 100 μM) at 37°C and 5% CO_2_. After 24 h, each sample was centrifuged (300xg, 5 min) and culture supernatants were collected and frozen immediately at -80°C for no longer than three weeks until the cytokine levels were measured (as specified above). Appropriate leukocyte pellets were stained with anti-TLR4 mAbs (see Flow cytometry). All chemicals needed were purchased from Sigma-Aldrich (Steinheim, Germany), unless otherwise specified.

### Flow cytometry

100 μl of fresh heparinized blood or 100 μl of leukocyte pellets from ex vivo culture were stained with 10 μl of PE-anti-TLR4 mAbs (MCA2061PE) for 30 min in darkness (RT). An isotype-matched control antibody was used to detect nonspecific staining (MCA929PE) and mouse anti human CD14 mAbs—for the monocyte gating strategy (MCA1568F). All antibodies were purchased from AbD Serotec, Düsseldorf, Germany. Next, erythrocytes were lysed through incubation with the lysing solution (Becton Dickinson) for 10 min at RT; the cells were washed twice with PBS containing 1% FCS and resuspended with 0.5 ml of 1% paraformaldehyde solution. Thirty thousand events were acquired (FACSCalibur, BD, Biosciences, Mountain View, CA) and monocytes were gated according to their characteristic FSC/SSC profiles compared with the CD14 dot plot. The TLR4 level was analyzed with the CellQuestPro software, ver. 6.0, BD) and measured as Δ mean fluorescence intensity (MFI): ΔMFI = MFI_TLR4_-MFI_isotype_.

### Data analysis

Normality of variables was tested with the Kolmogorov-Smirnov test. Normally distributed continuous variables were expressed as mean ± SD, and categorical variables were summarized as a median with an interquartile range. Quantitative variables with normal distribution were analyzed with one-way or two-way ANOVA with Tukey HSD as a post-hoc test. Comparisons between groups with categorical variables were evaluated by Kruskal-Wallis followed by Dunn test. In each group, correlation analyses between continuous or categorical variables were performed with Pearson’s or Spearman’s tests, respectively. In whole study group, a univariate analysis was performed to determine the relationship between the outcome variable (NAFLD) and the independent clinical and study parameters. The predictors of the outcome parameter that significantly correlated in the univariate analysis were then included in a multiple stepwise linear regression model to verify the association between NAFLD and independent variables. A univariate analysis with TLR4 as an outcome variable was performed separately in NAFLD and non-NAFLD subjects in order to compare associations of TLR4 and the study variables between these two groups. We also presented the results of multiple linear regression analysis for TLR4 as a dependent variable (ex vivo studies). The efficacy of the regression model was evaluated with coefficient R^2^ and an adjusted R^2^. The accuracy of the predictors was estimated with standard error of the estimate (SEE). Data were analyzed using STATISTICA software version 7.1 (StatSoft. Inc., Tulsa, OK, USA) and *P* value ≤ 0.05 was considered statistically significant.

## Results

### Anthropometric, metabolic, and biochemical characteristics of the subjects

Table 1 summarizes the general characteristics of the subjects included in our study. The mean age was similar in all groups, however group I was dominated by women (68%), while group III—by males (54%).

**Table 1:** Baseline clinical, anthropometric, and biochemical characteristics of the study populations.

Parameters	Healthy control	Nonobese+NAFLD-MetS	Obese+NAFLD+MetS	*P* values between groups		
	group I	group II	group III			
	n = 22	n = 20	n = 28	I and II	I and III	II and III
Age (years)	47.0±6.2	49.1±6.8	51.9±10.7	ns	ns	ns
Gender (M:F)	7:15	10:10	15:13	ns	0.03	ns
Weight (kg)	59 (55–71.5)	65.1 (51.2–74.0)	104.9 (95–120)	ns	0.0001	0.0001
Waist (cm)	80.7±6.6	82.4±8.3	114.8±10.8	ns	0.003	0.004
BMI (kg/m^2^)	21.4±2.4	22.0±1.6	36.5±5.8	ns	0.0001	0.0002
hs-CRP (mg/dl)	2.6±0.5	3.17±1.3	7.33±2.8	ns	0.001	0.03
ALT (IU/L)	17.6±3.6	75.0±29.9	80.67±18.75	0.0001	0.0001	ns
AST (IU/L)	16.5±3.7	69.4±10.0	72.9±15.5	0.001	0.0001	ns
Glucose (mg/dl)	85.6±6.6	91.6±4.8	103.9±4.8	ns	0.0001	0.002
Insulin (μl/ml)	8.2±1.8	16.1±2.3	20.6±7.8	0.013	0.0001	0.032
C-peptide (ng/ml)	3.1±0.55	5.0±0.45	4.69±1.9	0.0001	0.0009	ns
HbA1C (%)	4.4±0.32	4.58±0.47	5.9±1.0	ns	0.0001	0.001
HOMA-IR	1.75±0.29	3.26±0.7	5.2±1.5	0.006	0.0001	0.006
Total cholesterol (mg/dl)	178.3±6.5	213.3±26.9	240.1±29.5	0.0009	0.0001	ns
LDL-cholesterol (mg/dl)	96.3 (88–95)	119.9 (99.5–128)	145.2 (126–165)	0.02	0.0001	0.000
HDL-cholesterol (mg/dl)	74.1±11.2	45.4±6.6	43.9±9.1	0.002	0.0001	ns
Triglyceride (mg/dl)	126.9±12.0	188.0±31.5	218.8±42.1	0.0004	0.0001	ns
Systolic BP (mmHg)	155.9±5.4	128.1±5.5	136.9±7.2	ns	0.0005	0.0009
Diastolic BP (mmHg)	76.9±5.1	77.7±5.9	81.9±6.6	ns	0.004	0.02
FLI	15.0 (9.4–26.3)	95.6 (88.2–98.0)	97.2 (94.4–98.7)	0.001	0.001	ns
HSI	28.4±1.8	46.3±5.1	48.5±6.9	0.001	0.001	ns
FIB-4	0.956±0.31	1.58±0.54	2.835±1.4	ns	0.001	0.002
IL-1β (pg/ml)	1.23±0.52	4.49±1.9	9.83±2.0	0.008	0.001	0.01
IL-6 (pg/ml)	-	3.66±1.1	6.72±1.75	-	-	0.03
TNFα (pg/ml)	3.02±0.5	16.5±1.0	27.7±3.6	0.001	0.0003	0.01
TLR4 (ΔMFI)	17.3±3.5	39.9±3.4	53.19±6.7	0.006	0.00001	0.01

concentrations of circulating IL-6 levels in healthy subjects were below the detection threshold.

Obese NAFLD individuals (group III) characterized by the significantly highest levels of hs-CRP (7.33±2.8 mg/dl), fasting glucose (103.9±3.8 mg/dl), HbA1C (5.9±1.0%), LDL-cholesterol (145.2 (126–165) mg/dl), both systolic and diastolic blood pressure (136.9±7.2, 81.9±6.6 mmHg respectively), as well as all the serum cytokines examined. Additionally, in this group, we noted significant positive correlations between IL-6 and TG (r = 0.32. *P*≤0.01) and glucose (r = 0.23, *P*≤0,035) as well as between TNFα and BMI (r = 0.42, *P*≤0.003), cholesterol (r = 0.36, *P*≤0.01), TG (r = 0.39, *P*≤0.001), insulin (r = 0.41, *P*≤0.021), and IL-6 (r = 0.43, *P*≤0.001). The TLR4 level on blood monocytes positively correlated with BMI (r = 0.34, *P*≤0.03) and TNFα (r = 0.39, *P≤*0.032). Moreover, the patients in this group met all IDF/NHLBI/AHA-2009 criteria of metabolic syndrome. Prediabetes was revealed by high glucose and HbA1C levels, which positively correlated with IL-1β; r = 0.52, *P*≤0.01 and r = 0.47, *P*≤0.022, respectively.

Based on the AU results as well as HSI (>36.0) and FLI (>60.0) levels, the participants of group II were also classified as NAFLD; however, they met only two of the five IDF/NHLBI/AHA-2009 criteria (HDL-cholesterol and triglyceride concentrations). These nonobese patients differed from the subjects included in group I in the significantly lower level of hs-CRP (3.17±1.3 mg/dl), glucose (91.6±4.8 mg/dl), insulin (16.1±2.3 μl/ml), HbA1C (4.58±0.47%), HOMA-IR (3.26±0.7), LDL-cholesterol (119.9 (99.5–128) mg/dl) and both systolic and diastolic BP (128.1±5.5 and 77.7±5.9, respectively). While the cytokine and monocyte TLR4 levels were statistically lower than in the participants of group III, they still exceeded the levels noted in the healthy volunteers (group I) (Table 1). Significant positive correlations were observed between IL-6 and TG (r = 0.39, *P*≤0.03), cholesterol (r = 0.43, *P*≤0.028) and IL-1β (r = 0.95, *P*≤0.0001) as well as between the TLR4 level and IL-1β (r = 0.34, *P*≤0.031) and TNFα (r = 0.33, *P*≤0.042). Although we noted statistical differences in the FIB-4 values between groups II and III (*P*≤0.002), as well as between groups III and I (*P*≤0.001), the FIB-4 indexes did not exceed the value of 3.25, which could indicate a high risk of liver fibrosis and need for liver biopsy (Table 1).

Data were expressed as means ± SD (normally distributed continuous variables) or as medians with interquartile ranges (categorical variables) (Kolmogorov-Smirnov test). Comparisons between groups were evaluated by one-way ANOVA with Tukey HSD as a post-hoc test (variable with normal distribution) or by Kruskal-Wallis followed by Dunn test (categorical variables). (ns—statistically not significant).

To determine the association between NAFLD and parameters indicated by the univariate correlation coefficients in all subjects (Table 2) we performed a stepwise multiple linear regression analysis.

**Table 2:** Univariate correlation analysis among NAFLD and related clinical and studied variables in all subjects (n = 70).

Variables	r	*P*
Age (years)	0.212	0.01
Waist (cm)	0.645	0.0001
BMI (kg/m^2^)	0.646	0.0001
hs-CRP (mg/dl)	0.346	0.001
ALT (IU/L)	0.750	0.0001
AST (IU/L)	0.739	0.0001
Glucose (mg/dl)	0.499	0.0002
Insulin (μl/ml)	0.644	0.0001
HbA1C (%)	0.543	0.0001
Total cholesterol (mg/dl)	0.728	0.0001
LDL-cholesterol (mg/dl)	0.673	0.0001
HDL-cholesterol (mg/dl)	-0.696	0.0001
Triglyceride (mg/dl)	0.703	0.0001
Systolic BP (mmHg)	0.392	0.003
Diastolic BP (mmHg)	0.287	0.009
IL-1β (pg/ml)	0.454	0.004
IL-6 (pg/ml)	0.472	0.002
TNFα (pg/ml)	0.536	0.0001
TLR4 (ΔMFI)	0.746	0.0001

According to this multiple linear regression analysis, HDL was significantly negative, while BMI, ALT, TG, TNFα and TLR4 were significantly positive contributors to NAFLD (Table 3).

**Table 3:** Stepwise multiple linear regression analysis with NAFLD as a dependent variable performed in all subjects (n = 70).

Independent variables	beta	t	*P*
BMI (kg/m^2^)	0.349	3.830	0.03
HDL-cholesterol (mg/dl)	-0.220	-2.420	0.01
ALT (IU/L)	0.396	4.282	0.001
Triglyceride (mg/dl)	0.115	1.264	0.037
TNFα (pg/ml)	0.288	3.616	0.001
TLR4 (ΔMFI)	0.180	1.942	0.02
R^2^ = 0.915; R^2^ adj. = 0.855; SEE = 11.8			

(beta—standardized regression coefficient)

Additionally, we performed another univariate correlation analysis in order to demonstrate possible differences between NAFLD and non-NAFLD subjects in the context of association of TLR4 and the studied variables (Table 4).

**Table 4:** Comparison of univariate correlation analysis of the blood monocytes TLR4 level and the studied variables between non-NAFLD (n = 22) and all NAFLD (n = 48) subjects.

Variables	non-NAFLD subjects		NAFLD subjects	
	r	*P*	r	*P*
BMI (kg/m^2^)	0.207	0.38	0.343	0.03
HOMA-IR	0.053	0.82	0.378	0.016
TNFα (pg/ml)	0.268	0.08	0.840	0.036

As can be seen from the above table (Table 4), the level of blood monocyte TLR4 in the NAFLD patients strongly correlated with BMI, HOMA-IR, and TNFα while these correlations in healthy subjects were insignificant.

### Determination of the TLR4 level on monocytes of whole blood cultures

**Fig 1:**
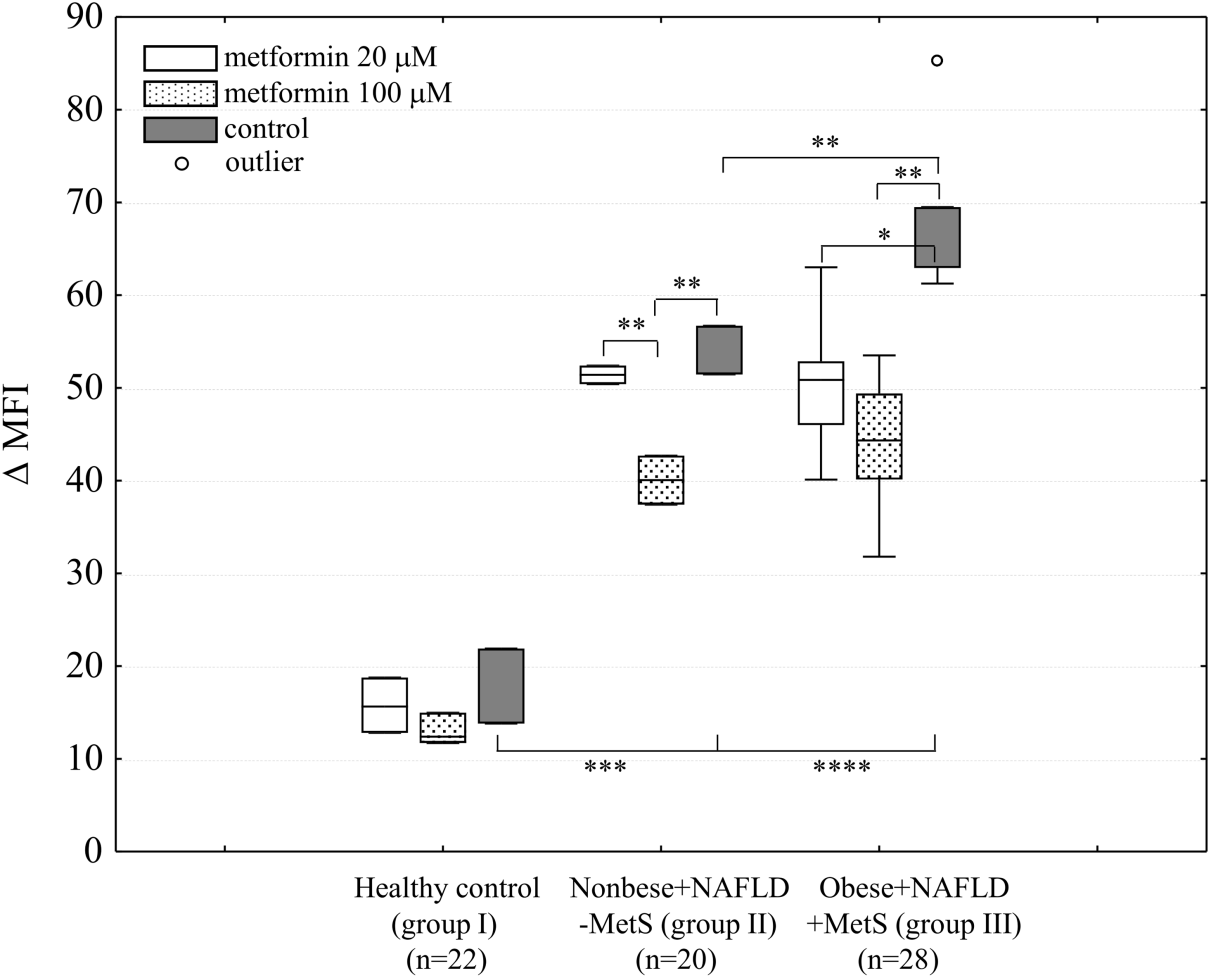
Metformin (20 μM or 100 μM) decreases TLR4 level (ΔMFI) on monocytes in cultures of subjects’ whole blood. Data from flow cytometry analysis showing that the greatest reduction of TLR4 level was noted on monocytes derived from obese subjects with NAFLD and MetS (*P*≤0.001). Data are expressed as medians with interquartile ranges. Group I—healthy individuals, group II—nonobese NAFLD without MetS, group III—prediabetic, obese NAFLD with MetS. *P≤0.05, **P≤0.01, ***P≤0.001, ****P≤0.0001 (Kruskal-Wallis followed by Dunn test). ΔMFI = MFI_TLR4_-MFI_isotype_.

We determined the membrane TLR4 level on monocytes identified from whole blood ex vivo cultures with or without the metformin (20 μM or 100 μM) treatment. A 24-h time point was chosen based on our earlier studies with whole blood cultures stimulated with LPS, which showed that a maximum decrease in the production of pro-inflammatory cytokines was observed 24 h after metformin treatment (S2 Table). We found that the TLR4 level on metformin-untreated monocytes significantly varied depending on the health status (*P*≤0.001, ANOVA Kruskal-Wallis test) (Fig 1), and corresponded to the results obtained for fresh blood monocytes (Table 1). The metformin treatment, especially at its higher concentration (100 μM), significantly decreased the TLR4 level: in group II—from ΔMFI = 51.2±8.5 to 41.0±3.2 (by 19.9±3.8%, *P*≤0.04), and in group III—from ΔMFI = 68.7±8.4 to 44.5±10.2 (by 35±5.9%, *P*≤0.001) (Fig 1).

### Production of pro-inflammatory cytokines in whole blood cultures

Consequently, the next aim of our work was to find the relationships between the monocyte TLR4 level, metformin effects, cytokine production, and health status of the participating individuals. We measured the concentration of IL-1β, IL-6, and TNFα in supernatants of ex vivo whole blood cultures.

**Fig 2:**
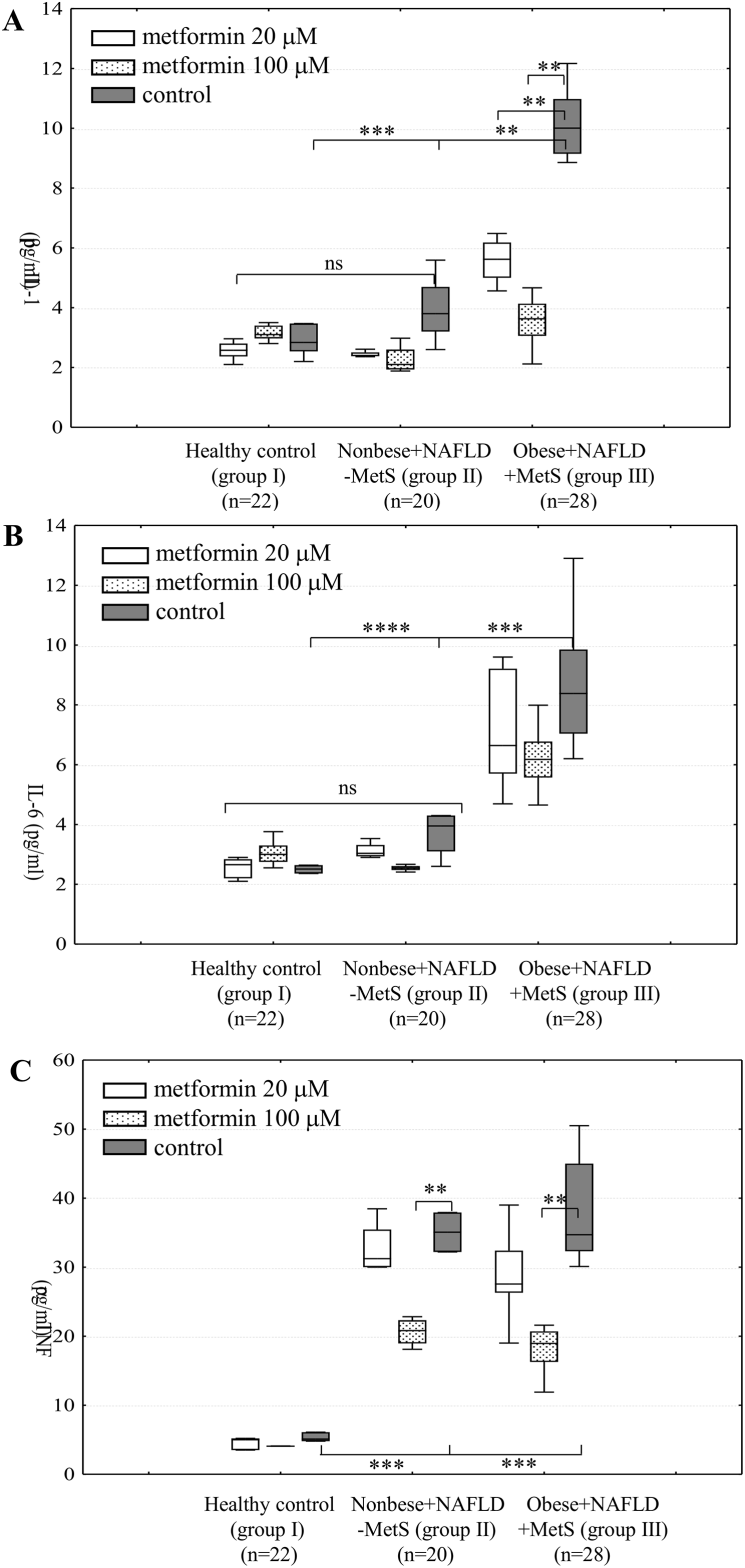
Metformin (20 or 100 μM) influences pro-inflammatory cytokine production in cultures of subjects’ whole blood. Data from ELISA showing that only leukocytes derived from prediabetic, obese patients with NAFLD and MetS (group III) responded to the metformin treatment with decreased production of all studied cytokines: IL-1β (A), IL-6 (B), and TNFα (C). Higher concentration of metformin (100 μM) significantly reduced TNFα levels in blood cultures originated from nonobese NAFLD (group II). I group represents healthy volunteers. Data are expressed as medians with interquartile ranges. (ns—statistically not significant when compared both inter- and intragroup). **P≤0.01, ***P≤0.001, ****P≤0.0001 (Kruskal-Wallis followed by Dunn test).

In the supernatants of metformin untreated whole blood culture, the highest levels of IL-1β and IL-6 were indicated for the obese NAFLD patients, which differed significantly from the other two groups (*P*≤0.01–0.0001 depending on the group and cytokine) (Fig 2A and 2B). Only these participants’ monocytes responded to both metformin concentrations with lowered IL-1β production. Regardless of the presence of metformin, we did not observe any significant differences in the IL-1β and IL-6 levels among the individuals of the other two groups (Fig 2A and 2B).

In contrast to the cytokines described above, we found that whole blood cultures originating from participants of both II and III group were significant sources of TNFα (*P*≤0.001 in comparison to healthy controls) (Fig 2C). The metformin treatment (only at the higher concentration) resulted in a decrease in the TNFα production to comparable levels in blood cultures derived from all NAFLD patients—regardless of the presence of obesity (*P*≤0.01) (Fig 2C). However, we noted that the reduction of the TNFα level differed in the particular groups: in samples derived from group II, the TNFα concentration was decreased by 29.2±5.8%, and in those from group III– 57.8±8.9%.

We found correlations between cytokine and TLR4 levels in the particular groups. In the case of the metformin untreated blood samples from group III, we noted a strong positive correlation between the receptor and IL-1β (r = 0.7, *P*≤0.02) and TNFα (r = 0.77, *P*≤0.003) levels. Addition of metformin (100μM) slightly decreased the r-value, which was still significant: r = 0.66, *P*≤0.034 and r = 0.59, *P*≤0.021 for IL-1β and TNFα, respectively. In samples originating from group II, we found a positive correlation only between the TLR4 level and the TNFα concentration—r = 0.81, *P*≤0.01 –which also was flattened in the presence of metformin (100μM)–r = 0.54, *P*≤0.021.

In summary, to find the main determinants of the effect of metformin on TLR4 a stepwise multiple linear regression analysis was performed in all subjects (Table 5). The results indicated that before ex vivo metformin treatment the monocyte TLR4 level was strongly associated with NAFLD (*P*≤0.023) and TNFα (*P*≤0.01) while after the treatment these significances were lowered to *P*≤0.05 and *P*≤0.09, respectively.

**Table 5:** Stepwise multiple linear regression analysis with TLR4 as a dependent variable performed in all subjects before and after ex vivo metformin (100 μM) treatment.

Independent variables	without metformin			with metformin		
	beta	t	*P*	beta	T	*P*
NAFLD	0.611	2.373	0.023	0.461	2.330	0.05
TNFα	0.250	2.828	0.01	0.111	0.933	0.09
	R^2^ = 0.883; R^2^ adj. = 0.804; SEE = 17.5			R^2^ = 0.803; R^2^ adj. = 0.780; SEE = 15.8		

(beta—standardized regression coefficient)

## Discussion

The present study evaluated the response of newly diagnosed NAFLD patients with or without obesity to the metformin effect in the context of the monocyte TLR4 level and production of pro-inflammatory cytokines. We found that metformin decreased the TLR4 level and pro-inflammatory response in all NAFLD patients; however, its efficacy depended on their health and phenotype status at the beginning of the study, in particular the presence of obesity. A better beneficial effect of metformin was observed in the prediabetic, obese individuals with NAFLD and MetS than in the nonobese NAFLD patients. Specifically, in ex vivo experiments metformin significantly attenuated the relationships between TLR4, NAFLD, and TNFα; however, in vivo studies should be performed to confirm this association.

A number of reports underline inflammation as a link between obesity, NAFLD, and MetS where it is considered as a cause rather than a consequence of steatosis [3]. One of the clinically useful indicators of systemic inflammation is hs-CRP—a protein produced predominantly by the inflamed liver, but also by adipose tissue [23]. According to Kushner et al., an hs-CRP level over 10 mg/dl reflects clinical, while 3–10 mg/dl—subclinical inflammation [24]. Indeed, in our studies, all patients had an elevated concentration of this protein, which in the case of the obese subjects positively correlated with their BMI. The highest hs-CRP level, although not exceeding 10 mg/dl, was noted in the obese NAFLD individuals, suggesting both adipose tissue and liver as a source of this protein. Since their serum levels of IL-1β, IL-6, and TNFα were also the highest among the studied participants, we could not eventually exclude a low-grade inflammatory state. Additionally, the positive correlation between IL-1β and the glucose level or HbA1C indicated a high risk of T2DM development [4]. Although the prevalence of NAFLD is more common among obese subjects, the disease may also be present in a nonobese population [25]. In patients with NAFLD, visceral adiposity influences the manifestations of MetS but not the severity of liver damage [26]. Indeed, in our investigations, the obese NAFLD patients met all the IDF/NHLBI/AHA-2009 criteria of MetS and the nonobese patients met only two, whereas liver cell necrosis (measured as ALT values) was comparable in both groups of the subjects.

Our results of the multiple stepwise regression analysis of the whole study group indicated BMI, ALT, TG, TNFα, and TLR4 as positive and HDL as negative contributors to NAFLD. Thus, these findings support previous studies [27–29]. However, including TLR4 into this group of factors is a novel observation. Moreover, we also demonstrated strong positive correlations between blood monocyte TLR4 levels and BMI, HOMA-IR as well as TNFα among all NAFLD patients (some of them prediabetic) in contrast to the healthy volunteers. Similar correlations were described by Dasu et al. [30] in recently diagnosed T2DM subjects. Additionally, interesting results were presented by De Mallo et al., where TLR4 expression in peripheral blood mononuclear cells was reduced in overweight individuals with metabolic syndrome after weight loss [31].

Since it is expressed on blood cells, mainly monocytes, TLR4 is a potent activator of innate immunity when stimulated by gut-derived lipopolysaccharide (LPS) or FFAs. Activated cells are then a source of pro-inflammatory signals and promote inflammation after recruitment into adipose tissue and liver. Additionally, the fact that high glucose enhances TLR4 expression on human monocytes [32] and its higher activity reflects an increasing risk of co-morbidities in patients with MetS [33] raises the question about a beneficial effect of the decreasing TLR4 level. Therefore, the next part of our studies focused on the ex vivo metformin effect on the TLR4 level and cytokine production in blood samples originating from all the subjects studied. Although metformin has been lately described as an anti-inflammatory agent [15, 16] only two papers of Soraya et al. link attenuated TLR2 and TLR4 activity with protection of infarcted hearts in rats treated with this drug [17, 18]. In humans after oral administration of metformin in a therapeutic dose, its plasma concentration reached 10–40 μM, and in portal vein– 40–80 μM [34]. Although rapidly eliminated from plasma, metformin is accumulated in erythrocytes and is then slowly released back [35]. Therefore, these cells represent most likely a secondary compartment of its distribution [36]. In other studies, Lalau et al. revealed that, after metformin administration, its plasma concentration in patients ranged 0–113 μg/ml while erythrocyte levels– 0–61 μg/ml [37]. Therefore, in our experiments, we chose two final metformin concentrations: 20 μM (corresponding to 2.5 μg/ml) and 100 μM (corresponding to 12.5 μg/ml).

Our findings demonstrated that metformin significantly decreased the TLR4 level on monocytes originating from all NAFLD patients in a dose-dependent manner; however, its efficacy depended largely on the presence of obesity. Obesity is associated with increased activation of blood monocytes [38], and one of the contributing mechanisms is the extracellular signal-regulated kinases (ERK)1/2 pathway. As indicated by Arai et al., metformin inhibits ERK1/2 activation [16]; however, further studies are indispensabe to clarify other mechanisms influenced by this drug. At present, the anti-inflammatory potential of metformin is well documented [15, 16]. Also in our study, metformin decreased TNFα production in the samples of all patients; however, reduced levels of IL-1β and IL-6 were observed exclusively in blood cultures derived from obese patients with NAFLD and MetS. This may result from the prediabetic phenotype of these individuals [39]. Therefore, such activity of metformin resulting in an IL-1β decrease could be promising in early anti-diabetic therapy in obese NAFLD patients [4].

The final stepwise multiple regression analysis revealed a strong effect of metformin on attenuation of the link between TLR4, TNFα and NAFLD. Those observations confirm the beneficence of the metformin treatment since TLR4 contributes to the development of NAFLD and MetS [5, 40].

Summarizing our main results, we demonstrated that the blood monocyte TLR4 level positively correlated either with NAFLD or obesity; prediabetic, obese patients with NAFLD and MetS were characterized by the highest level of this receptor. We also presented novel findings that, by attenuation of the blood monocyte TLR4 level, metformin could reduce their inflammatory potential—critical after recruitment of these cells into liver. However, this finding should be confirmed after in vivo metformin administration.

## Supporting Information

S1 Table Patient's evaluation questionnaire. (PDF)

S2 Table Ex vivo production of pro-inflammatory cytokines by blood leukocytes of healthy donors after metformin and/or LPS treatment. (PDF)
