# Supplementary material for: Metformin Changes the Relationship between Blood Monocyte Toll-Like Receptor 4 Levels and Nonalcoholic Fatty Liver Disease—Ex Vivo Studies
Source: PLoS One. 2016 Mar 1;11(3):e0150233. doi: 10.1371/journal.pone.0150233 (PMC4773077; doi:10.1371/journal.pone.0150233)
Supplement: S1 Table — (PDF) [file pone.0150233.s001.pdf]

S1 Table. Patient's evaluation questionnaire.

	**Date:**	
Initials		
Age		
Sex	F	M

Medical history

Alcohol consumption	<20g/day	>20 g/day
Cigarettes smoking	YES	NO

Have you got any of the following disorders?

Hypertension	YES	NO
Diabetes mellitus	YES	NO
Prediabetic state such as impired fasting glucose or impaired glucose tolerance	YES	NO
Dyslipidemia: hypercholesterolemia or high triglycerides?	YES	NO
Hepatitis type B	YES	NO
Hepatitis type C	YES	NO
Autoimmune hepatitis	YES	NO
Hemochromatosis	YES	NO
Wilson's disease	YES	NO
Other storage diseases	YES	NO
Severe liver leisure/liver cirrhosis	YES	NO
Serious kidney disease or renal failure	YES	NO
Neoplasm	YES	NO
Hematologic diseases	YES	NO
Serious diseases of cardiovascular system/atherosclerosis	YES	NO
Other serious diseases?		

Metformin		
Glucocorticosteroids		
Chemotherapeutics		

Cytostatic drugs
NSAID
Other drugs:

Additional results

Weight(kg)
Height(cm)
BMI (kg/m2)
Waist circumference (cm)
Hips circumference (cm)
WHR
SBP (mmHg)
DBP(mmHg)
Hs-CRP (mg/dl
Fasting glucose (mg/dl)
Fasting insulin (µl/ml)
C-peptyd (ng/ml)
HBA1C%
Total cholesterol(mg/dl)
LDL (md/dl)
HDL (mg/dl)
Triglycerydes (mg/dl)
ALT (IU/L)
AST (IU/L)
FZ ALP (IU/L)
GGT GGTP (IU/L)
HBSAg
Anti-HCV antibodies
ANA
Iron (ug/dl)
Ferritin (ng/ml)
Transferin
Total protein (g/dl)
Albumin (g/dl)
PT (min)
INR
HGB
RBC
WBC
PLT (10^9/L)

OGTT (75 g of glucose)	Fasting glucose (mg/dl)	Glucose after two hrs (mg/dl)

Indexes

HOMA-IR: (glucose*insulin):22.5
FIRI=insulin [µIU//mL] × glucose (mmol/L)/25

FLI= (e0.953*loge (triglycerides) + 0.139*BMI + 0.718*loge (GGT) + 0.053*waist circumference – 15.745)/(1 + e0.953*loge (triglycerides) + 0.139*BMI + 0.718*loge (GGT) + 0.053*waist circumference – 15.745) * 100

HSI= 8*ALAT/ASPAT ratio + BMI (+2 if female)

FIB4= Age (years) × AST (U/L)/Platelet count (10^9/L) × [ALT (U/L)] ^{½}

Image studies

Ultrasound of the abdomen and/or CT of the abdomen

Liver biopsy

Kwestionariusz oceny Pacjenta

Data:

Inicjały		
Wiek		
Płeć	K	M

Wywiad Chorobowy

Spożycie alkoholu:	<20g/dobę	>20g/dobę
Palenie tytoniu	TAK	NIE

Czy występują u Pani/Pana następujące zaburzenia?

Nadciśnienie tętnicze	TAK	NIE
Cukrzyca	TAK	NIE
Stan przedcukrzycowy tj: nieprawidłowa glikemia na czczo lub upośledzona tolerancja glukozy	TAK	NIE
Zaburzenia lipidowe: Hipercholesterolemia i/lub Hipertrójglicerydemia	TAK	NIE
Wirusowe zapalenie wątroby typu B	TAK	NIE
Wirusowe zapalenie wątroby typu C	TAK	NIE
Autoimmunologiczne zapalenie wątroby	TAK	NIE
Hemochromatoza	TAK	NIE
Choroba Wilsona	TAK	NIE
Inne choroby spichrzeniowe	TAK	NIE
Ciężkie uszkodzenie wątroby/marskość wątroby	TAK	NIE
Poważna choroba lub uszkodzenie nerek	TAK	NIE
Choroba nowotworowa	TAK	NIE
Choroby hematologiczne	TAK	NIE

Ciężkie choroby układu sercowo-naczyniowego/miażdżyca	TAK	TAK
Inne ciężkie choroby (jakie?)		

Metformina
Glikokortykosteroidy
Chemioterapeutyki
Cytostatyki
NLPZ
Inne leki:

Badania dodatkowe

Waga (kg)
Wzrost (cm)
BMI (kg/m2)
Obwód talii (cm)
Obwód bioder (cm)
WHR
RR skurczowe (mmHg)
RR rozkurczowe (mmHg)
Hs-CRP (mg/dl)
Glikemia na czczo (mg/dl)
Insulina na czczo (µl/ml)
C-peptyd (ng/ml)
HBA1C%
Cholesterol całkowity (mg/dl)
LDL (md/dl)
HDL (mg/dl)
Trójglicerydy (mg/dl)
ALT (IU/L)
AST (IU/L)
FZ (IU/L)
GGT (IU/L)
HBSAg
Przeciwciała anty-HCV
ANA
Żelazo (ug/dl)
Ferrytyna (ng/ml)
Transferyna
Białko całkowite (g/dl)
Albumina (g/dl)
PT (min)
INR
HGB
RBC
WBC
PLT (10^9/L)

**Test OGTT** (75g glukozy)	Glukoza na czczo (mg/dl)	Glukoza po 2 godz: (mg/dl)

Wskaźniki

HOMA-IR: (glukoza*insulina):22.5
FIRI=insulina [µIU//mL] × glukoza (mmol/L)/25
FLI= (e0.953*loge (trójglicerydy) + 0.139*BMI + 0.718*loge (GGT) + 0.053*obwód pasa – 15.745)/(1 + e0.953*loge (trójglicerydy) + 0.139*BMI + 0.718*loge (GGT) + 0.053* obwód pasa – 15.745) * 100
HSI= 8*ALAT/ASPAT + BMI (+2 gdy kobiety)
FIB4= Wiek (lata) × AST (U/L)/liczba płytek (10^9/L) × [ALT (U/L)] ^{½}

Badania obrazowe

USG jamy brzusznej i/lub KT jamy brzusznej
Biopsja wątroby
