# Supplementary material for: Metformin Changes the Relationship between Blood Monocyte Toll-Like Receptor 4 Levels and Nonalcoholic Fatty Liver Disease—Ex Vivo Studies
Source: PLoS One. 2016 Mar 1;11(3):e0150233. doi: 10.1371/journal.pone.0150233 (PMC4773077; doi:10.1371/journal.pone.0150233)
Supplement: S2 Table — (PDF) [file pone.0150233.s002.pdf]

S2 Table. Ex vivo production of pro-inflammatory cytokines by blood leukocytes of healthy donors after metformin and/or LPS treatment.

Metformin/LPS	Cytokines (pg/ml)	Incubation time (h)	
		24	48
Metformin 20 μg/ml LPS 100 ng/ml	IL-1β	462 ± 53#	524 ± 40
	IL-6	456 ± 68	510 ± 28
	TNFα	406 ± 48#[a]	593 ± 58
Metformin 100 μg/ml LPS 100 ng/ml	IL-1β	561 ± 59#	590 ± 38
	IL-6	499 ± 41	607 ± 44
	TNFα	601 ± 32#	698 ± 42
LPS 100 ng/ml	IL-1β	721 ± 39*	662 ± 53
	IL-6	628 ± 41*	582 ± 39
	TNFα	836 ± 56*	720 ± 46
Metformin 20 μg/ml	IL-1β	2.4 ± 0.6	2.36 ± 0.6
	IL-6	2.5 ± 0.39	2.6 ± 0.7
	TNFα	3.8 ± 0.7	3.0 ± 0.42
Metformin 100 μg/ml	IL-1β	2.8 ± 0.59	2.7 ± 0.32
	IL-6	3.0 ± 0.45	2.81 ± 0.41
	TNFα	3.5 ± 0.5	3.1 ± 0.86
Control	IL-1β	2.6 ± 0.33	2.8 ± 0.42
	IL-6	2.2 ± 0.42	2.73 ± 0.54
	TNFα	4.3 ± 0.8	3.86 ± 0.66

Blood cell cultures of healthy volunteers (n=15) were treated with metformin (20 μg/ml or 100 μg/ml) and with or without LPS (100 ng/ml). After 24 h or 48 h of incubation time pro-inflammatory cytokines level was measured in culture supernatants. Data were expressed as mean ± SD. * statistically significant in comparison to untreated control cells, $P \leq 0.05$. # statistically significant in comparison to culture treated only with LPS, $P \leq 0.05$. [a] statistically significant in comparison to 48 h, $P \leq 0.05$ (one-way ANOVA followed by Tukey post-hoc, Wilcoxon paired test, STATISTICA software version 7.1).
